# Efficient Evaluation of Concrete Fracture Surface Roughness Using Fringe Projection Technology

**DOI:** 10.3390/ma16124430

**Published:** 2023-06-16

**Authors:** Meiling Dai, Xirui Wang, Cheng Cheng, Zhuoli Chen, Jiyu Deng

**Affiliations:** 1 School of Civil and Transportation Engineering, Guangdong University of Technology, Guangzhou 510006, China; meiling-dai@163.com (M.D.); 15859919698@163.com (X.W.); 1216416395cchh@gmail.com (C.C.); 18438596038@163.com (Z.C.); 2 School of Architecture and Urban Planning, Guangdong University of Technology, Guangzhou 510080, China

**Keywords:** fringe-projection technology, concrete fracture surfaces, roughness, fractal dimension

## Abstract

The evaluation of concrete surface roughness is crucial in the field of civil engineering. The purpose of this study is to propose a no-contact and efficient method for the measurement of the roughness of concrete fracture surfaces based on fringe-projection technology. A simple phase-correction method using one additional strip image is presented for the phase unwrapping to improve the measurement efficiency and accuracy. The experimental results indicate that the measuring error for plane height is less than 0.1mm, and the relative accuracy for measuring a cylindrical object is about 0.1%, meeting the requirements for concrete fracture-surface measurement. On this basis, three-dimensional reconstructions were carried out on various concrete fracture surfaces to evaluate the roughness. The results reveal that the surface roughness (R) and fractal dimension (D) decrease as the concrete strength increases or the water-to-cement ratio decreases, consistent with previous studies. In addition, compared with the surface roughness, the fractal dimension is more sensitive to the change in concrete surface shape. The proposed method is effective for detecting concrete fracture-surface features.

## 1. Introduction

The evaluation of the concrete surface roughness is an essential task in the field of civil engineering. Firstly, the roughness of the joint surface of precast concrete components significantly impacts the mechanical properties of the joint and can affect the shear capacity of the joint surface after assembly, which is closely related to structural safety [1,2,3,4,5,6,7]. Secondly, the surface roughness of old concrete directly affects the bonding performance of old and new concrete [8,9,10]. Furthermore, understanding the crack path and damage distribution on the fracture surface is crucial, and this can be achieved by analyzing the fracture characteristics of concrete specimens obtained through mechanical testing [11,12,13]. The fracture performance of concrete is closely related to the roughness of the fracture surface [14,15]. Moreover, the concrete surface roughness may affect the bond capacity of the near-surface-mounted Fiber-reinforced polymer [16]. Therefore, the measurement of concrete surface roughness holds great practical importance [17].

Currently, there are two main methods for detecting the roughness of concrete: contact and noncontact measurement methods [18,19]. Contact methods based on “line” sampling include mechanical probing [20], contour texturing [21], and processing of digital images (PDI) methods [22]. These methods are flexible and moderately priced, but their accuracy is poor, and they do not adequately reflect the distribution of roughness on the surface. Additionally, they tend to cause irreversible damage to the rough surface. Contact-inspection methods based on “surface” sampling include the standard plate-comparison method [23], sand-cone method [24], water-accumulation method, and sheet-layer-concrete island method. Among these methods, the sand-cone method is the most widely used. The process involves pouring a certain volume (V) of powdered material, usually fine sand, onto a dry, rough surface to be detected, spreading it evenly so that it covers the most projecting point of the rough surface, and then calculating the area (S) covered by the powdered material. The concrete surface-roughness index is then calculated by V/S [25]. However, the sand-cone method is also accompanied by some shortcomings and inconveniences. The detection surface must be kept horizontal, a secondary cleaning of the rough surface may be necessary, and the accuracy of the detection results is greatly affected by the shape and particle size of the powder material used [26]. In addition, it is impossible to realize the digitization and informatization of the detection process and results.

Noncontact methods for detecting concrete surface roughness include laser scanning, photogrammetry, circular-texture instruments, and binocular-vision methods [18,19]. Among them, laser triangulation ranging is widely used in concrete surface measurement. The basic principle of this method is to project a laser beam onto a surface at an angle and then detect and analyze the reflected light [27]. However, laser triangulation ranging uses a line-acquisition method, which requires multiple scans to complete the rough surface acquisition. Therefore, obtaining a dense point cloud can be time-consuming.

Different from the laser-scanning method, fringe-projection technology is an area-scanning three-dimensional measurement technique that uses a projector and a camera based on the triangulation principle [28,29]. The recommended four-step phase-shifting method is used for phase calculation to ensure accuracy and efficiency. However, the phase values obtained by the arctangent algorithm are truncated within (−π, π) or (0, 2π), so an unwrapping algorithm is necessary to obtain a continuous phase distribution. Currently, there are two main categories of unwrapping algorithms: spatial phase unwrapping and temporal phase unwrapping [30,31]. The spatial phase-unwrapping method finds an optimal phase-unwrapping path in the phase map and uses methods such as the branch-cut method and quality-map-guided method [32,33,34]. The advantage of the spatial phase-unwrapping method is that only one wrapped-phase map is needed for phase-unwrapping calculations. However, this method may result in errors for objects with discontinuous surfaces or sharp height changes, as the fringes are prone to break and are discontinuous, causing the fringe order to jump during phase unwrapping. On the other hand, the temporal multi-frequency method projects a series of sinusoidal fringe patterns with different frequencies, and the obtained multiple wrapped-phase images are used to assist phase unwrapping [35]. This method makes up for the shortcomings of the spatial method and ensures the accuracy of fringe order in phase unwrapping. However, it requires the projection and collection of multi-frequency fringe patterns. For example, in the four-step phase-shifting method, at least eight images need to be projected and collected for each measurement, resulting in a relatively low measurement efficiency.

The objective of this study is to propose a noncontact and efficient method for measuring concrete surface roughness using fringe-projection technology. The limitations of existing methods are addressed by introducing a point-by-point scanning approach and incorporating a simple correction strategy for phase unwrapping. By reducing the number of projected fringe patterns and improving the accuracy of phase measurement, the method significantly enhances measurement efficiency. Experimental results demonstrate the effectiveness and reliability of the method.

## 2. Materials and Methods

### 2.1. Concrete Specimens

Concrete specimens with different water-to-cement ratios of 0.54, 0.46, and 0.38 were produced and subjected to testing. The raw materials used included cement (OPC 42.5, Guangzhou Shijing Cement Company, Guangzhou, China), river sand, water, and coarse aggregate with lengths between 5 mm and 15 mm. Six cubic specimens with dimensions of 150 mm × 150 mm × 150 mm were produced for each water-to-cement ratio. Three of them were used for compression tests, and the other three were used for splitting tensile tests. The mix proportions of the concrete specimens are shown in Table 1.

### 2.2. Definition of Roughness

The concrete surface roughness [36], *R*, is defined as the ratio of the real surface area, $A_{t}$, to the projected surface area, $A_{p}$:

(1) $$R=A_{t}/A_{p}$$

**Figure 1:**
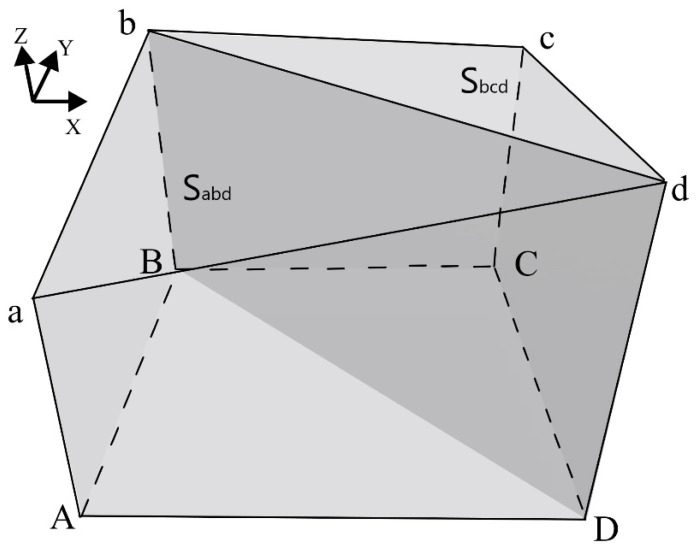
The area unit. A, B, C, and D are the projection points on the XY plane corresponding to the spatial points, a, b, c, and d.

The real area of the concrete surface is calculated using the concept of an area unit [34]. This means that the spatial quadrilateral composed of two adjacent coordinate points in every two adjacent rows is a unit. As shown in Figure 1, “*abcd*” is an area unit, and the area of the unit is equal to the sum of the areas of two triangles, i.e., $S_{abcd}=S_{abd}+S_{bcd}$. The area of the whole surface can be obtained by adding the area of the spatial quadrilateral formed by all points on the surface, i.e., $A_{t}=\Sigma s_{1}+\Sigma s_{2}$.

In this study, the area of a spatial triangle is calculated using Heron’s formula:

(2) $$S=\sqrt{p(p-l)(p-m)(p-n)}$$

where $p=\frac{l+m+n}{2}$, and *l*, *m*, *n* are the lengths of the three sides of the spatial triangle, which can be calculated from the spatial coordinates of the three points.

As shown in Figure 1, the projection of the spatial unit “*abcd*” on the XY plane is “*ABCD*”, so the projection area is the area of the planar quadrilateral $S_{ABCD}$. For the entire real surface, the projection area is $A_{p}=\Sigma S_{ABCD}$. The surface roughness can then be calculated through Equation (1).

**Figure 2:**
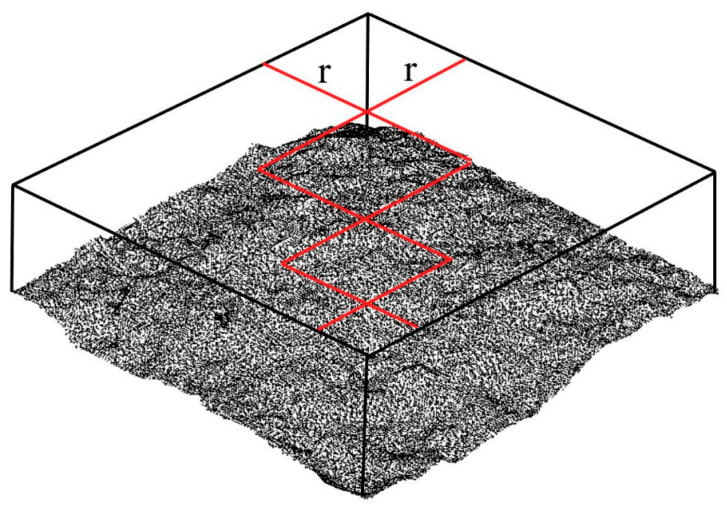
Projective covering method. The red line represents the size of the unit.

**Figure 3:**
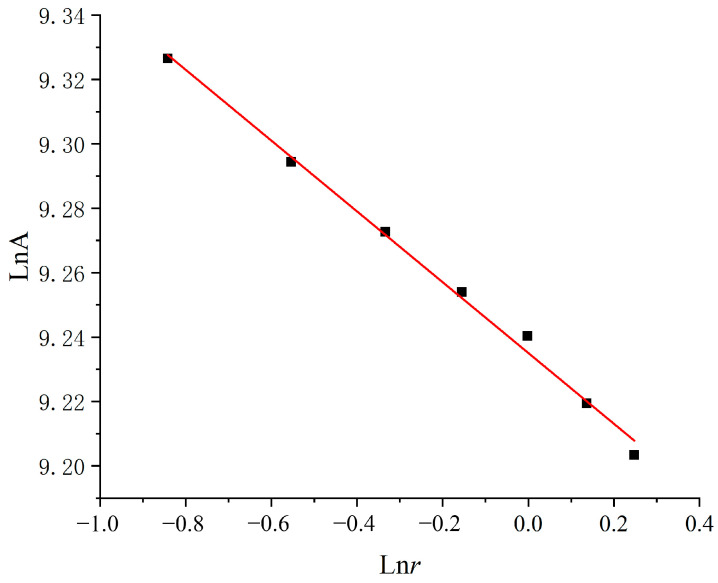
The lnA-ln*r* relationship.

In general, the fracture surface of concrete has fractal characteristics, and the surface roughness is related to the fractal dimension. Therefore, the fractal dimension in fractal theory is also used to quantify the surface roughness of concrete fracture surfaces. The fractal dimension can be calculated by using the projection-covering method [37], which is similar to the box-counting method, as shown in Figure 2 and Figure 3. The fractal dimension, D, is determined by the rate at which the apparent area, A, changes as the size of the units, *r*, decreases. The equation is described as

(3) $$D=2-\underset{r\to 0}{\lim}\frac{lnA(r)}{lnr}$$

where *r* is the size of the varying area unit, as shown in Figure 2; A(*r*) is the total area of the surfaces obtained when *r* is the size of the area unit.

The areas, A(*r*), and the corresponding unit size, *r*, are plotted on a logarithmic scale and fitted by linear regression, as shown in Figure 3. The good linear relationship indicates the fractal characteristic of the concrete surface, and the fractal dimension value can be obtained from the slope of the LnA-ln*r* curve. Equation (3) can be rewritten as

(4) $$D=2+\left|\frac{lnA(r)}{lnr}\right|$$

### 2.3. Measuring Principle

In fringe-projection 3D measurement systems, a digital projector is utilized to project a sequence of sinusoidal fringe patterns onto the surface of the object. A camera captures the deformed fringe patterns caused by variations in the height of the object’s surface. Phase-shifting and phase-unwrapping algorithms are then employed to demodulate the fringe phase from the deformed fringe patterns. Next, the height of each point on the object surface is obtained through a phase-height calibration process based on two reference planes. Finally, a horizontal calibration process is carried out to convert the image coordinates to world coordinates. To ensure the accuracy of phase unwrapping, a simple correction method is proposed by projecting an additional strip image during the phase demodulation process.

#### 2.3.1. Phase Calculation

- Four-step phase-shifting method

When the sinusoidal fringe patterns are projected onto an object’s surface, the variations in surface height cause lateral displacement of the fringes, which can be obtained through phase shifting of the fringe patterns point-by-point [28]. In this paper, the four-step phase-shifting method is used for phase calculation due to its insensitivity to nonlinear errors. This method involves projecting four fringe patterns with phase-shift values of 0, π/2, π, and 3π/2, which can be expressed as intensities using Equations (5)–(8).

(5) $$I_{1}(x,y)=a(x,y)+b(x,y)sin[\varphi (x,y)]$$

(6) $$I_{2}(x,y)=a(x,y)+b(x,y)sin[\varphi (x,y)+\pi /2]$$

(7) $$I_{3}(x,y)=a(x,y)+b(x,y)sin[\varphi (x,y)+\pi ]$$

(8) $$I_{4}(x,y)=a(x,y)+b(x,y)sin[\varphi (x,y)+3\pi /2]$$

In these equations, *a*(*x*, *y*) represents the background, *b*(*x*, *y*) relates to the reflectivity of the object’s surface, and *φ*(*x*, *y*) is the phase to be retrieved. The phase and background can be obtained from Equations (9) and (10), respectively.

(9) $$\varphi (x,y)=arctan\frac{I_{1}(x,y)-I_{3}(x,y)}{I_{2}(x,y)-I_{4}(x,y)}$$

(10) $$a(x,y)=\frac{I_{1}+I_{2}+I_{3}+I_{4}}{4}$$

However, the phase map, φ(x,y), obtained from Equation (9) is wrapped in the range of −π~π, so it must be unwrapped before using it to calculate the height of the object surface. Assume that $\emptyset^{u}\left(x,y\right)$ is the unwrapped phase; the relationship between the wrapped phase $\varphi \left(x,y\right)$ and the unwrapped phase is

(11) $$\emptyset^{u}\left(x,y\right)=\varphi (x,y)+2\pi \cdot N(x,y)$$

where $N\left(x,y\right)$ is the fringe order.

- Phase unwrapping:

Quality-guided phase-unwrapping method unwraps the phase by using a quality map to guide the phase-unwrapping path [38]. In this study, the phase-derivative variance is used as the phase quality map. It is defined as follows:

(12) $$q_{m,n}=\frac{\sqrt{\sum_{i=m-k/2}^{m+k/2}\sum_{j=n-k/2}^{n+k/2}{\left(\Delta^{x}{}_{i,j}-\bar{\Delta^{x}{}_{m,n}}\right)}^{2}}+\sqrt{\sum_{i=m-k/2}^{m+k/2}\sum_{j=n-k/2}^{n+k/2}{\left(\Delta^{y}{}_{i,j}-\bar{\Delta^{y}{}_{m,n}}\right)}^{2}}}{k^{2}}$$

where *k* is the size of the square window centered at (*m*, *n*), $\Delta_{i,j}^{x}$ and $\Delta_{i,j}^{y}$ are the phase gradient in the *x* and *y* directions, and ${\bar{\Delta }}_{i,j}^{x}$ and ${\bar{\Delta }}_{i,j}^{y}$ are the average values of the phase gradient in the *k* × *k* window centered at (*m*, *n*). According to this quality map, the phase-unwrapping process starts from high-quality points to low-quality points until it is finished. Although some unwrapping errors may remain undetected and propagate, this method is surprisingly robust in practice [39].

Because the concrete surface is basically continuous, the quality-guided phase-unwrapping method is sufficient for the concrete surface measurement. However, the quality maps change corresponding to different object surfaces, so the unwrapping paths for the reference planes and different concrete surfaces are usually different, which leads to confusion in fringe orders in Equation (11), causing erroneous results in the following phase-height calibration. This problem may be solved by using the multi-frequency fringe-projection method, which requires at least one additional set of phase-shifting fringe patterns to be projected. For the four-step shifting method, at least eight fringe patterns are needed for the unwrapped-phase calculation.

In this study, a simple fringe-order correction method is proposed to adjust the initially obtained unwrapped phase using the quality-guided phase-unwrapping method, thereby obtaining the correct unwrapped phase. This method involves projecting only one additional strip-marker image, through which a fixed fringe order can be detected and used for phase correction. In measurement, the strip-mark image and the four-step phase-shifting sinusoidal fringe patterns are projected onto the object’s surface. By using a total of five images, the acquisition efficiency is improved, and the accuracy of the roughness measurement for concrete surfaces is ensured.

The strip-mark image is designed as a black-and-white image with white background and one black strip, whose position corresponds to one fringe order ($N_{cc}$) of the sinusoidal fringes projected.

The initial fringe order can be calculated after phase unwrapping by using the quality-guided method.

(13) $$N(x,y)=\left[\frac{\emptyset^{u}\left(x,y\right)-\varphi (x,y)}{2\pi }\right]$$

where N(x,y) represents the fringe-order map before correction, $\emptyset^{u}\left(x,y\right)$ is the initial unwrapped phase from the quality-guided method, and $\varphi \left(x,y\right)$ denotes the wrapped phase.

The actual fringe order corresponding to the marked fringe is $N_{cc}$, so the correction value of the fringe order is ${N_{c}=N}_{cc}-N$. Thereby, the initial fringe-order map from Equation (13) can be corrected as

(14) $$N_{cc}(x,y)=N(x,y)+N_{c}$$

where, $N_{cc}(x,y)$ is the corrected fringe-order map.

Finally, the unwrapped phase is modified as below.

(15) $$\emptyset_{c}^{u}\left(x,y\right)=N_{cc}\left(x,y\right)\cdot 2\pi +\varphi \left(x,y\right)$$

where, $\emptyset_{c}^{u}\left(x,y\right)$ is the corrected unwrapped phase, it will be applied for the 3D shape reconstruction.

#### 2.3.2. Phase-Height Calibration

**Figure 4:**
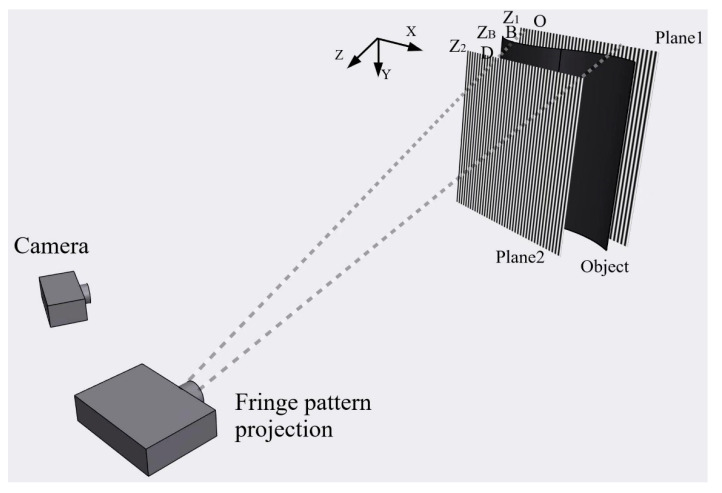
Two reference-planes-based phase-height calibration.

**Figure 5:**
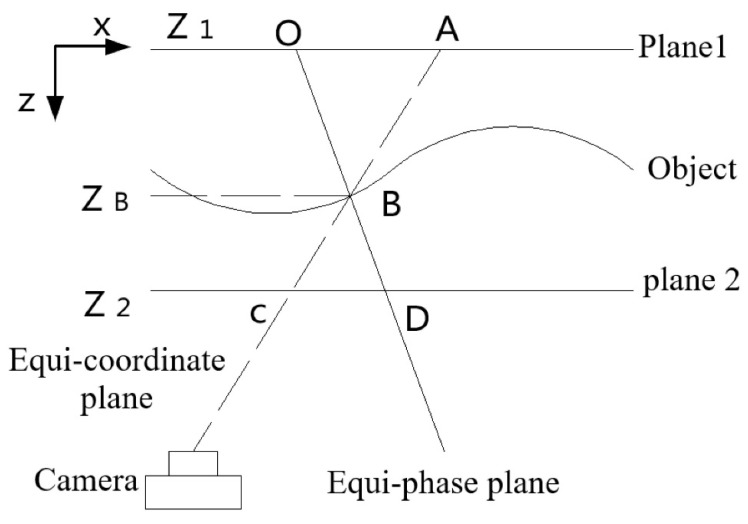
Top view of the calibration system.

The process of converting phase information of the object’s surface into depth or height information is called phase-height or depth calibration. In this paper, the absolute height of the object’s surface will be calculated using the equal-phase coordinate method based on dual reference planes [28]. As shown in Figure 4 and Figure 5, the system includes two parallel reference planes, a projector for fringe projection, and a camera for recording the deformed fringe patterns. The distance h between the two planes is artificially introduced and precisely measured. Figure 4 shows the three-dimensional view of the calibration system, and Figure 5 exhibits the corresponding top view.

When a sinusoidal fringe’s intensity is distributed along the *x*-axis, the phase value increases monotonically along the *x*-axis. At a fixed *x*-coordinate, the phase remains constant along the *y*-axis, and the projection plane from the projector with the same phase is referred to as the equiphase plane. This plane is perpendicular to the *x*-*z* plane, and, as depicted in Figure 5, the object’s surface lies between two reference planes. The intersections of the equiphase plane with Plane 1, Plane 2, and the object are denoted by *O*, *D*, and *B*, respectively. Plane 1’s height, *Z*_1_, is 0, and Plane 2’s height, *Z*_2_, is *h*. Therefore, the height of the object surface point *B*, *Z*_B_, is determined using the triangle similarity theorem:

(16) $$\frac{\bar{OA}}{\bar{CD}}=\frac{Z_{B}-Z_{1}}{Z_{2}-Z_{B}}=\frac{Z_{B}}{h-Z_{B}}$$

Here, $\bar{OA}=x_{A}-x_{O}$, and $\bar{CD}=x_{D}-x_{C}$, and $x_{A}$, $x_{O}$, $x_{D}$, $x_{C}$ are the image coordinates in the *x* direction of the points *A*, *O*, *D*, and *C*, respectively, as shown in Figure 5. Since $x_{A}=x_{C}=x_{B}$, we can derive that:

(17) $$Z_{B}=\frac{x_{B}-x_{O}}{x_{D}-x_{O}}\cdot h$$

For an object surface point *x_B_* with phase *ϕ*, we can locate identical phase value coordinates *x_O_* and *x_D_* on reference Plane 1 and Plane 2, respectively. *x_O_* or *x_D_* typically lies between two pixels rather than on a pixel. Therefore, we use linear interpolation to obtain the subpixel *x* coordinate. Thus, in Equation (17), *x_O_* and *x_D_* are of subpixel precision, while *x_B_* is an integer.

#### 2.3.3. Horizontal Calibration

After obtaining the height information of the object surface (the *Z* coordinates of the object surface points), the 3D measurement is converted into a 2D lateral measurement by converting the image coordinates to world coordinates [40].

Assuming that the image coordinate of a point on the object surface is (x,y), the corresponding homogeneous coordinate is (x,y,1), and the three-dimensional coordinate is (X,Y,Z), with its corresponding homogeneous coordinate being (X,Y,Z,1). Based on the principle of small-aperture imaging, the conversion relationship between image coordinates and world coordinates can be deduced using the following equation:

(18) $$\lambda \left[\begin{array}{c} x\\ y\\ 1 \end{array}\right]=\left[\begin{array}{cc} \begin{array}{cc} a_{x} & c\\ 0 & a_{y}\\ 0 & 0 \end{array} & \begin{array}{cc} u_{0} & 0\\ v_{0} & 0\\ 1 & 0 \end{array} \end{array}\right]\left[\begin{array}{cc} R & t^{T}\\ 0^{T} & 1 \end{array}\right]\left[\begin{array}{c} \begin{array}{c} X\\ Y \end{array}\\ \begin{array}{c} Z\\ 1 \end{array} \end{array}\right]$$

Here, λ is the scale factor, $\left(u_{0},v_{0}\right)$ is the image principal point, and $f_{u}=\frac{a_{x}}{dx}\;and\;f_{v}=\frac{a_{y}}{dy}$ represent the normalized focal length in the *x* and *y* directions, respectively. The tilt factor between the CCD axes is represented by *c*, which is generally small, ideally 0. The translation matrix is denoted by $t^{T}={\left[t_{1},t_{2},t_{3}\right]}^{T}$, and $R=\left[\begin{array}{ccc} r_{11} & r_{12} & r_{13}\\ r_{21} & r_{22} & r_{23}\\ r_{31} & r_{32} & r_{33} \end{array}\right]$ is the rotation matrix.

Equation (18) can be rewritten as:

(19) $$\left[\begin{array}{c} x\\ y\\ 1 \end{array}\right]=\left[\begin{array}{cc} \begin{array}{cc} p_{11} & p_{12}\\ p_{21} & p_{22}\\ p_{31} & p_{32} \end{array} & \begin{array}{cc} p_{13} & p_{14}\\ p_{23} & p_{24}\\ p_{33} & p_{34} \end{array} \end{array}\right]\cdot \left[\begin{array}{c} \begin{array}{c} X\\ Y \end{array}\\ \begin{array}{c} Z\\ 1 \end{array} \end{array}\right]$$

The coefficient matrix, $P=\left[\begin{array}{ccc} p_{11} & p_{12} & p_{13}\\ p_{21} & p_{22} & p_{23}\\ p_{31} & p_{32} & p_{33} \end{array}\begin{array}{c} p_{14}\\ p_{24}\\ p_{34} \end{array}\right]$, describes the transformation relationship between image coordinates and world coordinates and can be obtained through the camera calibration process. Equation (19) contains three equations, two of which are linearly independent, allowing the determination of the two unknowns, *X* and *Y*. For horizontal calibration, a reference plane with a checkerboard grid is adopted, and the image coordinates and corresponding world coordinates of the checkerboard grid corner points are used to determine the *P* matrix.

## 3. Results and Discussion

### 3.1. Validation

The accuracy of the concrete surface-roughness calculation depends on the measurement accuracy of its morphology. However, if the concrete fracture surface is irregular, it is difficult to determine the measurement accuracy. Thus, in this section, a flat plane and a standard cylinder with a diameter of 91 mm were measured, and the measurement error was analyzed to determine the precision of the proposed method.

**Figure 6:**
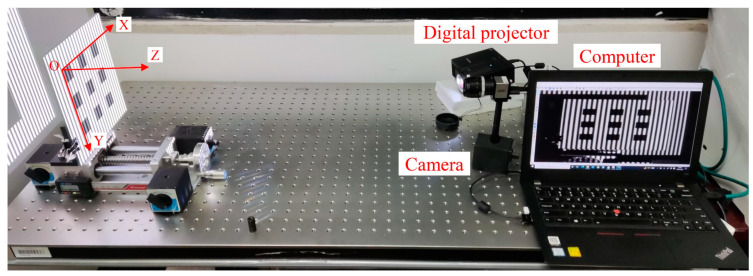
Experimental setup.

Figure 6 shows the experimental setup comprising a DLP projector (Ricoh, rw1120est, Tokyo, Japan) with a resolution of 1280 × 800 pixels, a 12-bit CMOS camera (IDS, 3370, Obersulm, Germany), a computer (ThinkPad, X11, Beijing, China), and a linear translation platform. The projector was employed to project fringe patterns generated by the computer, while the camera was used to capture the deformed patterns, and all these images were recorded by the computer. During the experiment, the grayscale of the projected fringe patterns was adjusted in the range of 50~250, ensuring that the projection-acquisition system exhibited a good linear response.

Prior to the measurement, the system required calibration. As illustrated in Figure 6, a calibration board with a checkerboard pattern (with a grid length of 25 mm) was vertically placed on the translation platform, perpendicular to the direction of movement (*Z*-axis). The *X*-axis of the world coordinate system was aligned with the horizontal axis on the calibration board, while the *Y*-axis was parallel to the vertical axis on the calibration board, as shown in Figure 6.

**Figure 7:**
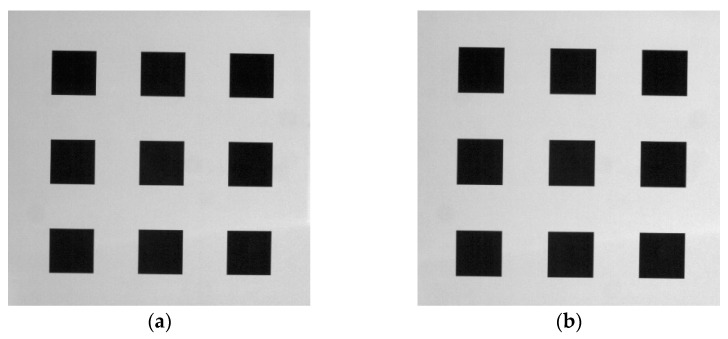
Background images of the calibration plate at (**a**) z = 0 mm and (**b**) z = 20 mm.

Initially, the calibration board was positioned at *Z* = 0 mm, and the four-step phase-shifting fringe patterns and a strip-marker map were projected onto it and captured. Next, the board was moved to *Z* = 20 mm, and the projection and capture processes were repeated. Positions *Z* = 0 and *Z* = 20 mm were designated as the two reference planes for height calibration (Section 2.3.2). Accordingly, the background images of the reference planes calculated using Equation (10) were utilized for the horizontal calibration (Section 2.3.3), as shown in Figure 7. The image coordinates of the corner points and their corresponding world coordinates were then substituted into Equation (19) to solve for the coefficient matrix *P*.

**Figure 8:**
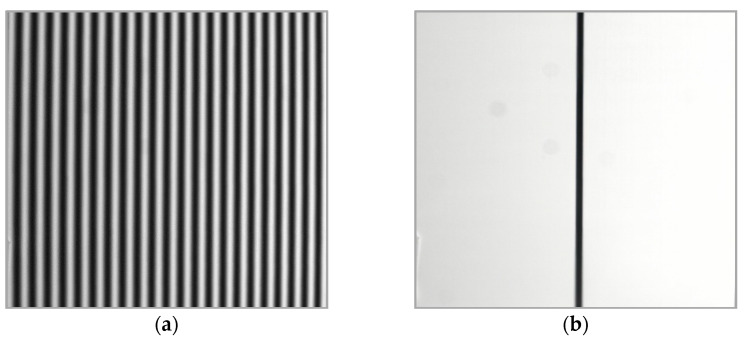
(**a**) Fringe pattern; (**b**) stripe marker.

To validate the phase-correction method proposed in Section 2.3.1, a flat plate was measured at different positions, including *Z* = 5, 10, and 15 mm. The measurement range of the plate was about 150 × 150 mm^2^, corresponding to 1500 × 1500 pixels, resulting in a scanning interval of 0.1 mm/pixel. Figure 8a,b depict one of the four-step phase-shifting fringe patterns and the strip-marker image captured, respectively. It should be noted that the position of the strip marker in black in Figure 8b corresponded to the 18th fringe of the projected fringes, thus $N_{cc}=18$.

**Figure 9:**
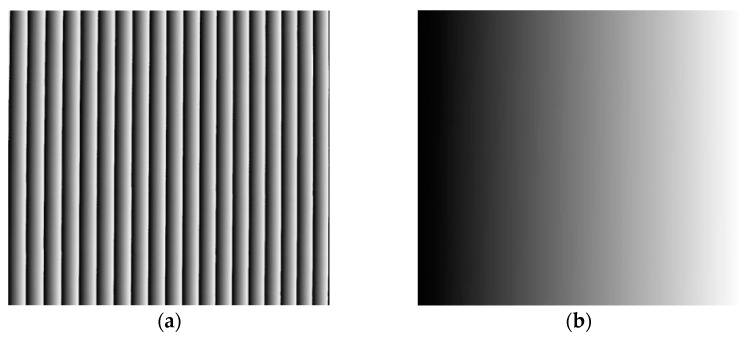
Phase before correction (**a**) the wrapped phase; (**b**) the initial unwrapped phase.

**Figure 10:**
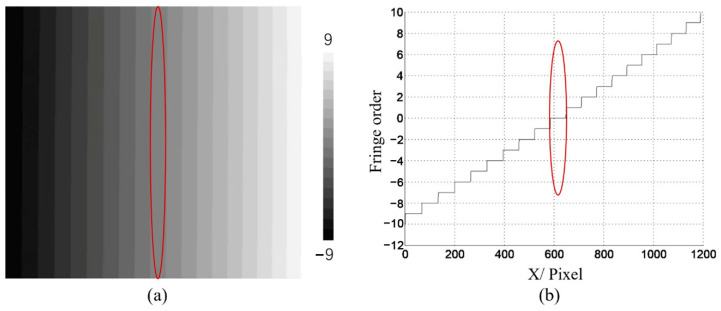
Fringe order before correction (**a**) the distribution map; (**b**) one row of (**a**). The red circle indicates the position of the stripe marker.

**Figure 11:**
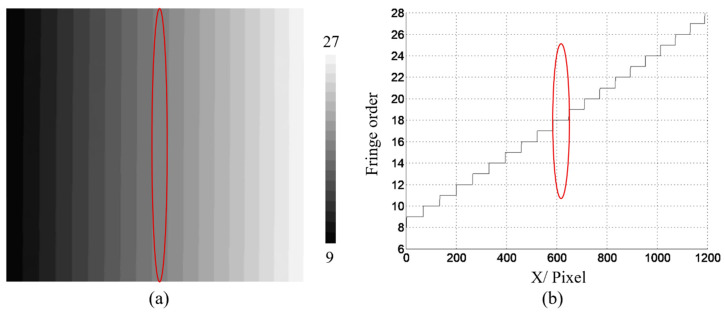
Fringe order after correction (**a**) the distribution map; (**b**) one row of (**a**). The red circle indicates the position of the stripe marker.

**Figure 12:**
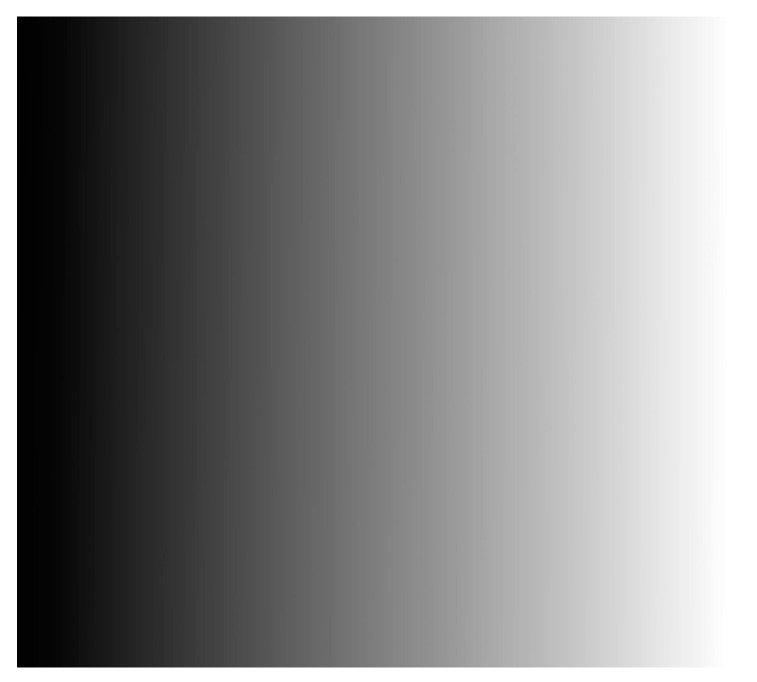
Modified unwrapped phase $\emptyset_{c}^{u}(x,y)$.

Figure 9a shows the wrapped phase $\varphi \left(x,y\right)$ obtained using the four-step phase-shifting method. Figure 9b displays the initial unwrapped-phase map $\emptyset^{u}(x,y)$ obtained using the quality-guided method. The initial fringe order $N\left(x,y\right)$ can then be obtained using Equation (13), as shown in Figure 10. Based on the strip-marker image, Figure 8b, the initial fringe order corresponding to the position of the strip marker was *N* = 0. However, the real fringe order corresponding to the position of the strip marker was $N_{cc}=18.$ Thus, the correction value of the initial fringe-order map $N\left(x,y\right)$ was obtained as $N_{c}=N_{cc}-N=18$ − 0 = 18. Thus, the initial fringe-order map $N\left(x,y\right)$ can be corrected as $N_{cc}(x,y)=N(x,y)+18$ (Equation (14)), as shown in Figure 11. Finally, the real unwrapped phase was calculated using Equation (15), as shown in Figure 12.

**Figure 13:**
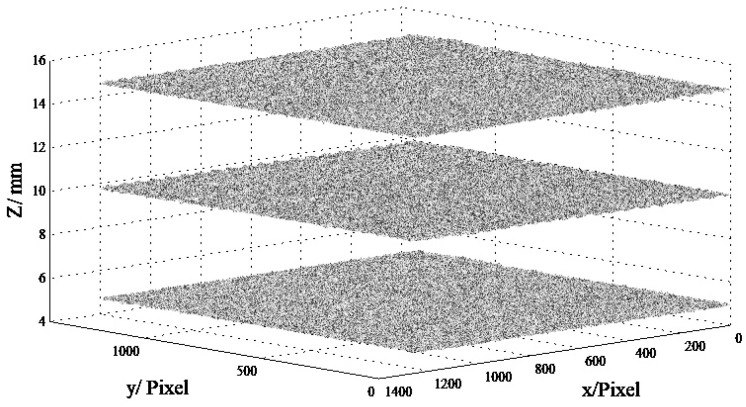
Height maps of planes at different positions.

Once the corrected unwrapped-phase maps $\emptyset_{c}^{u}(x,y)$ of the reference planes and the targets were obtained, the height calculation could be carried out using these phase maps (Section 2.3.2). Figure 13 shows the height measurement results of the planes at positions *Z* = 5, 1, and 15 mm. As observed, the measurement results for the three planes are 4.93 ± 0.033 mm, 10 ± 0.057 mm, and 14.95 ± 0.041 mm, respectively. The length of aggregate used in this study was in the range of 5~15 mm, indicating that the minimum characteristic size of the concrete fracture surface was at the millimeter level. Therefore, the measurement error was less than 0.1 mm, which met the requirements of concrete fracture-surface measurement [27].

**Figure 14:**
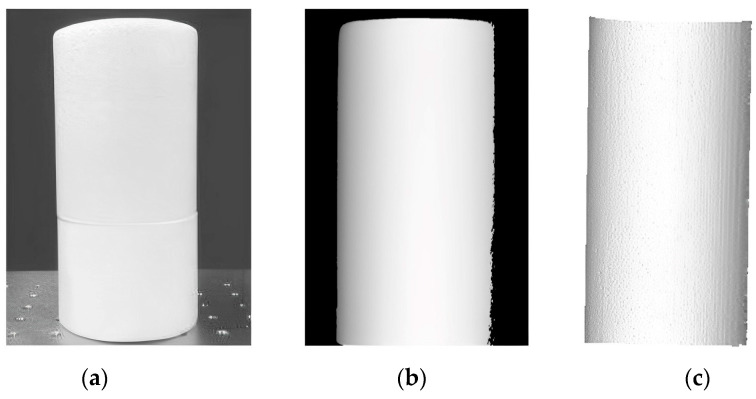
(**a**) A cylindrical object and the result: (**b**) height map; (**c**) 3D reconstructed surface.

To further verify the accuracy of the proposed method, a cylindrical object with a diameter of 91 mm was measured, as shown in Figure 14a. After the correct unwrapped phase was calculated using the proposed method, the height map of the cylinder was obtained from the phase-height calibration, as observed in Figure 14b. Then the 3D point data on the cylinder surface were calculated from Equation (19) using the height map and the matrix *P* obtained, and the reconstructed result is displayed in Figure 14c. The point data of several rows of the cylinder were fitted with the Equation: *f*(*x*_0_, *y*_0_, *D*) = (*x* − *x*_0_)^2^ + (*y* − *y*_0_)^2^ − (*D*/2)^2^ = 0, and the fitted diameter, *D*, was 91.1 mm, which was about 0.1% different from the ground truth 91 mm. This experiment further demonstrated the effectiveness of the proposed method in three-dimensional measurement. The results reveal that the proposed method can achieve high accuracy and meet the requirements of practical applications.

It should be noted that the measuring accuracy of the fringe-projection technology is also affected by the resolution of the camera and projector, ambient light, and lens distortion. By updating hardware and improving the measurement environment, the impact of the first two factors can be reduced. And the influence of lens distortion on measurement accuracy should be evaluated and corrected in future work.

### 3.2. Measurement of the Concrete Fracture Surface

According to the “Standard for Physical and Mechanical Properties Test Methods of Concrete” (GBT 50081-2019) [41], the compression test and splitting test were conducted using the universal testing machine (SINOTEST, YNS-Y3000, Changchun, China) at loading speeds of 0.5 MPa/s and 0.05 MPa/s, respectively. The compressive strength test and the splitting tensile test were conducted three times, respectively, and the average results were calculated and listed in Table 2.

**Figure 15:**
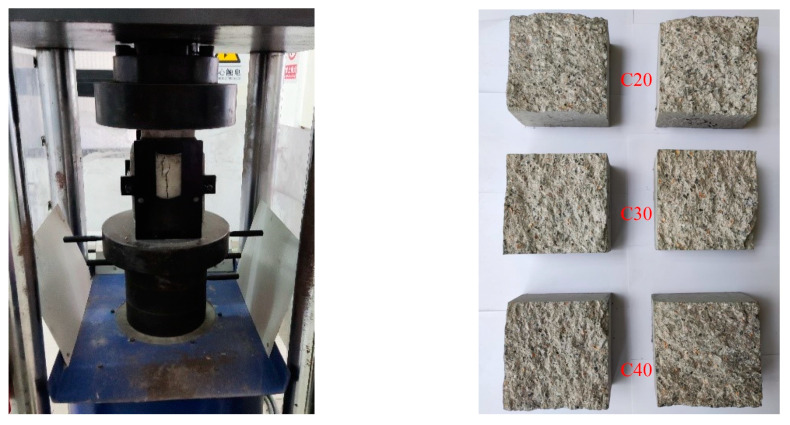
The splitting test and the fractured concrete.

For the splitting test, we prepared three specimens of each type of concrete material. However, due to inappropriate operation during the test or transportation, one to two specimens were damaged, resulting in incomplete concrete fracture surfaces. Therefore, in the measurement experiment, one specimen from each type, with a complete fracture surface, was selected for testing, as shown in Figure 15. Furthermore, the fracture behaviors of each type of concrete specimen were found to be similar in the splitting test. Thus, the roughness of the fracture surfaces of each type was assumed to be essentially the same.

**Figure 16:**
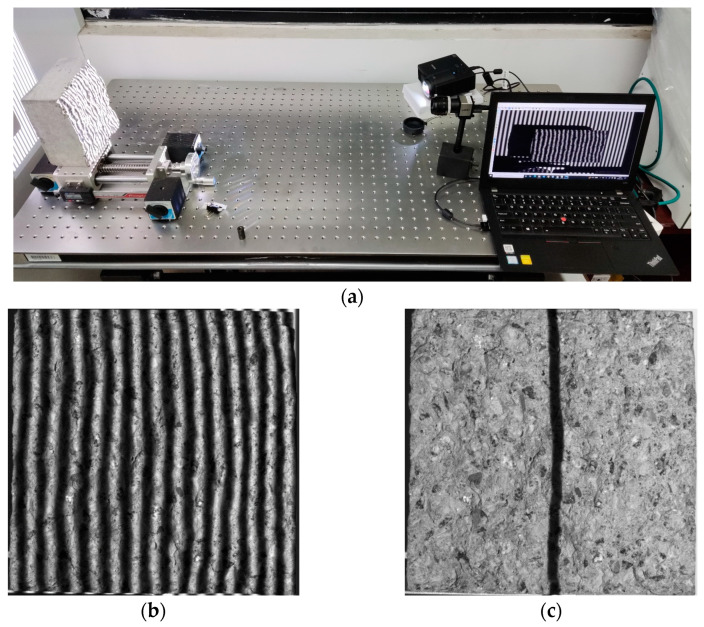
(**a**) Experiment site; (**b**) the deformed fringe pattern; (**c**) the strip marker.

Figure 16a displays the experiment site of the concrete surface measurement. Figure 16b,c show one of the fringe patterns and the strip-marker image captured. The location corresponding to the strip mark was marked in Figure 16b.

**Figure 17:**
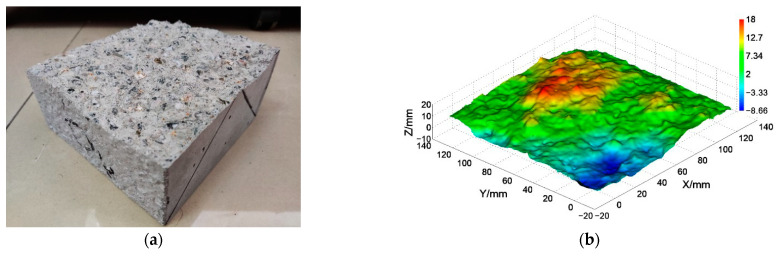
Concrete fracture surface from splitting testing (**a**) real surface; (**b**) reconstructed surface.

Figure 17a displays the rough surface of one concrete specimen, and its corresponding reconstructed surface is shown in Figure 17b. A comparison of the two figures reveals a good similarity between the ground truth and the reconstructed result, with all the details of the actual fracture surface evident in the reconstructed version. Using these measurement results, the surface roughnesses and fracture dimensions were calculated for all specimens, and the results are presented in Table 3.

**Figure 18:**
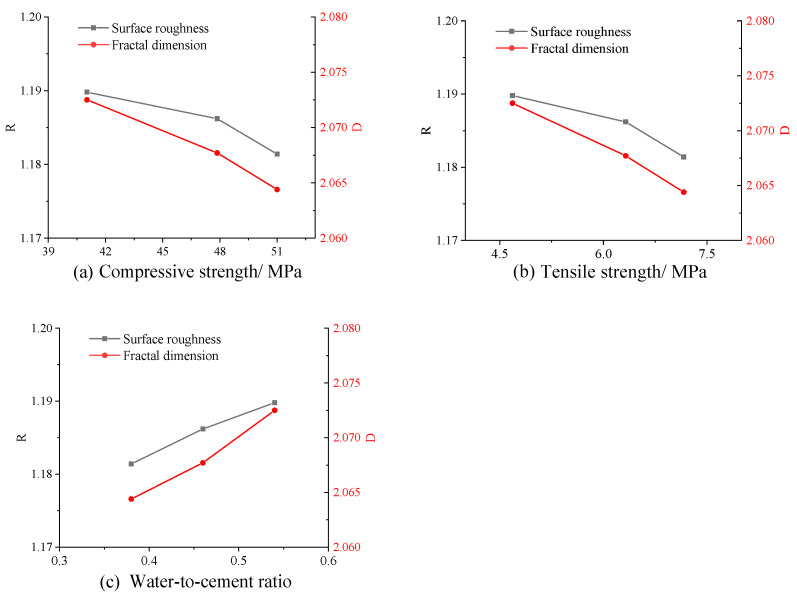
Measurement results of concrete fracture surfaces from splitting testing.

**Figure 19:**
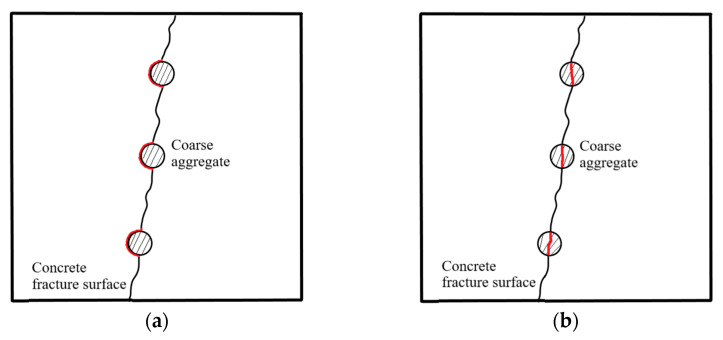
Two fracture paths in concrete materials: (**a**) pass around the coarse aggregate; (**b**) pass through the coarse aggregate.

Figure 18a,b depict the correlation between concrete surface roughness and its mechanical properties, i.e., compressive strength and tensile strength. Figure 18c presents the variation curve of concrete surface roughness with the water-to-cement ratio. Figure 19 exhibits two possible fracture paths in normal concrete materials [42]. Figure 19a illustrates that a crack travels around the aggregates, while Figure 19b represents that a crack passes through the aggregates directly. Generally, there are many factors that affect the fracture path in concrete materials [37,42], including water-to-cement ratio, coarse aggregate, additives, curing environment, and loading method. In this study, the water-to-cement ratio of the concrete specimens was in the range of 0.38~0.54, while the compressive strength was 40~50 MPa, and the tensile strength was 4~7 MPa. Meanwhile, the differences in the surface roughness (R) and fractal dimension (D) for different specimens were also very small. Nevertheless, a negative correlation was still observed between the surface roughness and mechanical properties (compressive strength and tensile strength), and a positive correlation was found between the surface roughness and water-to-cement ratio. This can be attributed to the fact that higher water-to-cement ratios cause a lower bond strength of cement mortar, which is more likely to lead to cracks forming along the edge of the coarse aggregate, resulting in rougher fracture surfaces. Conversely, lower water-to-cement ratios cause a higher bond strength of cement mortar, so cracks are more likely to pass through the coarse aggregate, resulting in smoother fracture surfaces. It is noted that either the surface roughness or the fractal dimension obtained in this study was a little lower than that obtained in the previous study [42]. This is mainly due to the different loading methods and different mix proportions, such as coarse aggregates and superplasticizers. The trends obtained in this study are consistent with the previous study, further validating the reliability and effectiveness of the proposed measurement method.

Furthermore, the rate of change for the fractal dimension (D) was observed to be faster than that of the surface roughness (R), indicating that the fractal dimension is more sensitive to changes in concrete surface shape than surface roughness. This suggests that the fractal dimension provides a more precise measurement of changes in concrete surface shape, as it takes into account the roughness at multiple length scales.

## 4. Conclusions

This paper proposes a concrete surface-roughness measurement method based on fringe-projection technology, which offers the advantages of noncontact, full-field, and point-by-point measurement over traditional methods. This method provides an effective means for detecting features on concrete surfaces. The main conclusions of this study are as follows:

(1) The fringe-projection system achieved a measuring accuracy for a plane height of less than 0.1 mm and a relative accuracy for measuring a cylindrical object of about 0.1%, meeting the requirements for concrete fracture-surface measurement.
(2) The unwrapped-phase correction method was experimentally validated and required only one additional strip image for fringe-order adjustment, ensuring measurement accuracy while improving efficiency.
(3) The measurement of concrete fracture surfaces revealed that higher concrete strength was found to correlate with decreased roughness and fractal dimension, while an increased water-to-cement ratio led to increased roughness and fractal dimension. This finding highlights the relationship between concrete fracture surfaces and their mechanical properties.
(4) In comparison to surface roughness, the fractal dimension is proved to be a more sensitive parameter for capturing changes in concrete surface shape.

As important postmortem evidence, the information on the concrete fracture-surface measurement helps people to understand the micromechanisms of fracture and plays an essential part in the evaluation of concrete material behavior. This study provides a noncontact and efficient method for concrete fracture-surface analysis. Furthermore, more factors affecting the roughness of the concrete fracture surface will be evaluated using the proposed method in future work.

## Figures and Tables

**Table 1:** Mix proportions (kg/m^3^).

Type	Water	Cement	Sand	Coarse
C20	210	388.9	630.7	1171.3
C30	210	456.5	606.7	1126.8
C40	210	552.6	573.09	1064.31

**Table 2:** Compressive strength and splitting tensile strength of concrete specimens.

Concrete Specimens	Water-to-Cement Ratio	Compressive Strength (MPa)	Splitting Strength (MPa)
C20	0.54	41.031 ± 0.599	4.684 ± 0.136
C30	0.46	47.859 ± 2.735	6.324 ± 0.165
C40	0.38	51.003 ± 1.546	7.164 ± 0.285

**Table 3:** Calculation results.

Concrete Specimens	Surface Roughness (R)	Fractal Dimension (D)
C20	1.1898	2.0725
C30	1.1862	2.0677
C40	1.1814	2.0644

## Data Availability

Not applicable.
